# Role of N6-methyladenosine in tumor neovascularization

**DOI:** 10.1038/s41419-024-06931-z

**Published:** 2024-08-05

**Authors:** Lu Zhao, Qinshan Li, Tongliang Zhou, Xuan Liu, Jing Guo, Qing Fang, Xiaoxue Cao, Qishun Geng, Yang Yu, Songjie Zhang, Tingting Deng, Xing Wang, Yi Jiao, Mengxiao Zhang, Honglin Liu, Haidong Tan, Cheng Xiao

**Affiliations:** 1 Institute of Clinical Medicine, China-Japan Friendship Hospital, Beijing, China; 2 China-Japan Friendship Hospital, Capital Medical University, Beijing, China; 3 Institute of Precision Medicine of Guizhou Province, Department of Obstetrics and Gynecology, Affiliated Hospital of Guizhou Medical University, Guiyang, Guizhou 550004 China; 4 Department of Clinical Biochemistry, School of Clinical Laboratory Science, Guizhou Medical University, Guiyang, Guizhou 550004 China; 5 Graduate School of Peking Union Medical College, Chinese Academy of Medical Sciences/Peking Union Medical College, Beijing, China; 6 China-Japan Friendship Clinical Medical College, Beijing University of Chinese Medicine, Beijing, China; 7 Department of Hepatobiliary Surgery, China-Japan Friendship Hospital, Beijing, 100029 China

**Keywords:** Tumour angiogenesis, Epigenetics

## Abstract

Tumor neovascularization is essential for the growth, invasion, and metastasis of tumors. Recent studies have highlighted the significant role of N6-methyladenosine (m^6^A) modification in regulating these processes. This review explores the mechanisms by which m^6^A influences tumor neovascularization, focusing on its impact on angiogenesis and vasculogenic mimicry (VM). We discuss the roles of m^6^A writers, erasers, and readers in modulating the stability and translation of angiogenic factors like vascular endothelial growth factor (VEGF), and their involvement in key signaling pathways such as PI3K/AKT, MAPK, and Hippo. Additionally, we outline the role of m^6^A in vascular-immune crosstalk. Finally, we discuss the current development of m^6^A inhibitors and their potential applications, along with the contribution of m^6^A to anti-angiogenic therapy resistance. Highlighting the therapeutic potential of targeting m^6^A regulators, this review provides novel insights into anti-angiogenic strategies and underscores the need for further research to fully exploit m^6^A modulation in cancer treatment. By understanding the intricate role of m^6^A in tumor neovascularization, we can develop more effective therapeutic approaches to inhibit tumor growth and overcome treatment resistance. Targeting m^6^A offers a novel approach to interfere with the tumor’s ability to manipulate its microenvironment, enhancing the efficacy of existing treatments and providing new avenues for combating cancer progression.

## Facts

- Neovascularization is a rate-limiting step in tumor progression.
- m^6^A modification participates in various aspects of cancer biology.
- Tumor neovascularization induces an immunosuppressive microenvironment.
- Immunosuppressive cells promote tumor neovascularization.

## Open Questions

- What is the role of m^6^A modification in different modes of tumor neovascularization and associated pathways?
- What is the relationship between m^6^A modification and anti-angiogenic drug resistance?
- Can anti-angiogenic therapy be combined with immunotherapy by targeting m^6^A regulators?
- Can m^6^A targeting effectively improve the limited efficacy of current anti-angiogenic therapy?

## Introduction

Tumor neovascularization ensures the acquisition of adequate oxygen and nutrients required for sustained tumor growth [1]. Notably, solid tumors tend to grow around blood vessels and cannot expand beyond 2 mm^3^ without vascularization [2, 3]. The induction of the “angiogenic switch”, which depends on a balance of angiogenic and anti-angiogenic factors, is a rate-limiting step in tumorigenesis, triggering exponential tumor growth [4, 5]. Neovascularization, considered as a hallmark of cancer, is indispensable for tumor proliferation, invasion, and metastasis [5]. Consequently, targeting tumor neovascularization has emerged as a crucial component of cancer therapy. Existing anti-angiogenic strategies primarily focus on the vascular endothelial growth factor (VEGF) or VEGF receptor (VEGFR) signaling pathway. Despite advancements, these approaches yield transitory benefits and often fail to achieve long-term clinical responses [6]. Increasing evidence indicates that tumor neovascularization is a complex process involving multiple components, underscoring the need to elucidate the underlying mechanisms to improve anti-angiogenic therapy efficacy [7–9].

Recently, researchers have proposed that non-mutational epigenetic reprogramming facilitates the acquisition of hallmark capabilities by tumors [10, 11]. Epigenetics refers to the study of heritable alterations that do not involve changes to the DNA sequence, including DNA and RNA methylation, nucleosome remodeling, and histone modifications [11, 12]. Over 170 RNA modifications have been identified, including N6-methyladenosine (m^6^A), 5-methylcytosine (m^5^C), N7-methylguanosine, and N1-methyladenosine [13]. The most prevalent RNA modification among these is m^6^A, first described in 1974, which occurs as an RNA methylation at the sixth nitrogen atom of adenosine [14]. m^6^A modification is a dynamic and reversible process that is installed by methyltransferases (“writers”), removed by demethylases (“erasers”), and recognized by RNA-binding proteins (“readers”) [15]. m^6^A participates in multiple aspects of RNA metabolism processes, including splicing, translation, stability, degradation, and nuclear export. It plays an essential role in reshaping the tumor microenvironment (TME), regulating cancer metabolism, and facilitating carcinogenesis [12, 15–17].

Recent evidence highlights the role of m^6^A in regulating tumor neovascularization. Our previous review linked m^6^A to immune reprogramming [16]. In this review, we aim to explore the regulatory role of m^6^A in tumor neovascularization, offering a comprehensive grasp of its significance in cancer therapy. This review introduces the diverse role of m^6^A in multiple modes of neovascularization and associated signaling pathways. Additionally, we concisely outline its contribution to vascular-immune crosstalk. Finally, we discuss the current development of m^6^A inhibitors and their potential clinical applications. This review clarifies the underlying mechanism of tumor neovascularization and provides novel insights into targeting m^6^A in anti-angiogenic therapy.

## Tumor neovascularization

**Fig. 1:**
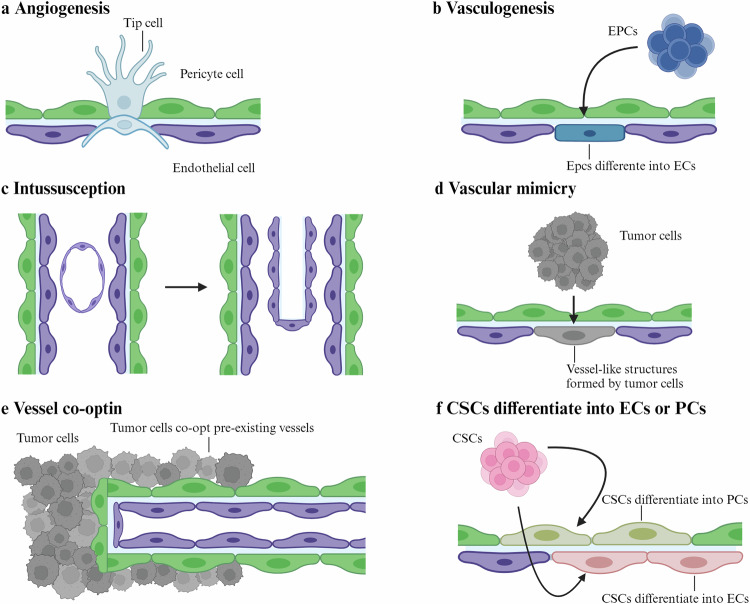
Modes of tumor neovascularization. There are several modes of tumor neovascularization. **a** Angiogenesis: blood vessels form from preexisting vessels through sprouting; **b** vasculogenesis: endothelial progenitor cells derived from the bone marrow are recruited and differentiate into endothelial cells to form blood vessels; **c** intussusception: transcapillary tissue pillars insert into the lumen of existing vessels, undergo vascular splitting, and eventually fuse to remodel the vascular network; **d** vascular mimicry: tumor cells form vessel-like structures; **e** vessel co-option: tumor cells hijack the existing vasculature and migrate along the vessel surface or infiltrate non-malignant tissues between vessels; **f** CSC differentiate into ECs or PCs: cancer stem cell differentiate into endothelial cells or pericytes. CSCs cancer stem cells, ECs endothelial cells, EPCs endothelial progenitor cells, PCs pericytes.

In 1971, Folkman proposed that solid tumor growth is always accompanied by the formation of new blood vessels, suggesting that inhibiting tumor vascularization could suppress tumor growth [2]. Since then, serveral modes of tumor neovascularization have been identified, including sprouting angiogenesis, vasculogenesis, intussusceptive angiogenesis, vasculogenic mimicry (VM), vessel co-option, and cancer stem cell (CSC)-derived vasculogenesis (Fig. 1) [7, 8]. The first three modes occur in both normal tissues and tumors, whereas the latter three are specific to tumor neovascularization. Among them, angiogenesis and VM are the most extensively studied.

### Angiogenesis

Angiogenesis is the traditional process by which new blood vessels form from preexisting vessels through sprouting. Initially, endothelial cells (ECs) loosen their junctions, increasing permeability and releasing plasma proteins. Subsequently, ECs sprout, with tip cells penetrating the basement membrane, and an imbalance between matrix metalloproteinase (MMP) and tissue inhibitor of metalloproteinase (TIMP) leads to extracellular matrix (ECM) degradation. Finally, ECs proliferate and migrate, accompanied by pericyte recruitment, resulting in the formation of new blood vessels [7, 18].

### Vasculogenic mimicry (VM)

The classical theory of tumor angiogenesis proposes that blood vessels are generated through ECs sprouting. However, in 1999, a paradigm shift occurred with the introduction of the concept of VM in a study focused on melanoma [19]. Unlike traditional angiogenesis, VM is independent of ECs which forms vessel-like structures by tumor cells [8]. Subsequent studies revealed that VM occurs not only in melanoma but also in glioma, hepatocellular carcinoma (HCC), and prostate cancer [20–22]. Mechanistically, VM involves the epithelial-mesenchymal transition (EMT) process and differentiation of CSCs [23, 24]. During VM, epithelial cell markers like E-cadherin are downregulated, whereas mesenchymal cell markers like VE-cadherin, vimentin are upregulated [24, 25].

## m^6^A components

### m^6^A writers

m^6^A writers are methyltransferases responsible for installing m^6^A and modifications on RNA.The core components include methyltransferase-like 3 (METTL3), methyltransferase-like 14 (METTL14), and Wilms tumor 1-associated protein (WTAP). METTL3 and its homolog METTL14 form an asymmetric heterodimer in a 1:1 ratio [26]. METTL3 functions as the catalytic subunit, whereas METTL14 acts as an allosteric activator, enhancing METTL3’s catalytic activity by providing an RNA-binding scaffold [27]. With the assistance of WTAP, an adapter subunit, the METTL3- METTL14 complex is located in nuclear speckles [28]. Additionally, RNA-binding motif protein 15 (RBM15) and its paralog RBM15B interact with WTAP-METTL3, recruiting it to proximal m^6^A consensus sites [29]. Other writer components, including vir-like m^6^A methyltransferase associated (VIRMA), zinc finger CCCH-type containing 13 (ZC3H13), and cbl proto-oncogene like 1 (CBLL1), also interact with WTAP [15]. Among them, VIRMA mediates m^6^A in the 3′ untranslated region (3′UTR) and near the stop codon by acting as a scaffold to hold WTAP/CBLL1/ZC3H13 together [30]. Methyltransferase-like 16 (METTL16) is an independent RNA methyltransferase that installs m^6^A on molecules such as U6 small nuclear RNA (snRNA), methionine adenosyltransferase 2A (*MAT2A*) mRNA, and metastasis-associated lung adenocarcinoma transcript 1(*MALAT1*) [31].

In various tumor types, METTL3 typically functions as an oncogenic factor, including acute myeloid leukemia (AML), lung cancer, HCC, among others, while exhibiting an anti-oncogenic role in endometrial cancer [32]. Additionally, METTL3 is implicated in therapy resistance across cancers. For instance, it promotes chemoresistance in HCC, breast cancer, and colorectal cancer (CRC), and reduces the effectiveness of immunotherapy in CRC and melanoma [33]. However, despite METTL14 lacking catalytic activity and functions as an allosteric activator of METTL3, its impact on tumors may not consistently align with METTL3. METTL14 exerts an oncogenic effect in HCC and AML, but suppresses tumor progression in CRC and renal cell carcinoma (RCC) [32, 34]. Additionally, decreased METTL14 has been reported to promote radiotherapy resistance in esophageal squamous cell carcinoma (ESCC) and sorafenib resistance in HCC [33]. This discrepancy could arise from the distinct target sites methylated by each protein within the writer complex. m^6^A methylation is preferentially enriched at DRACH (D = A, G, or U; R = G or A; H = A, C or U) motifs, suggesting numerous potential methylation sites exist within transcripts. However, the writer complex exhibits site- and transcript-specific selectivity, which may be influenced by transcription factors or histone marks that recruit the writer complex to specific genomic loci [15, 35].

### m^6^A erasers

The reversible nature of m^6^A modification is regulated by m^6^A erasers, which are limited in specific tissues and are context-dependent [35]. m^6^A demethylation is catalyzed by two enzymes, fat mass and obesity-associated (FTO) and AlkB homolog 5 (ALKBH5), both part of the ALKB enzyme family [36]. FTO is the first identified m^6^A eraser that is associated with obesity and energy homeostasis [37]. Initially, FTO was identified as a nucleus m^6^A demethylase, but later studies suggested its primary target might be N6,2′-O-dimethyladenosine (m^6^A_m_) rather than m^6^A [37]. m^6^A typically occurs at internal sites within mRNA, whereas m^6^A_m_ is located near the 5′cap structure. Subsequent studies indicated that FTO regulates m^6^A_m_ modification of snRNA, thereby affecting the alternative splicing of mRNA [38]. Despite a lower response rate, FTO can demethylate m^6^A and exhibit an oncogenic role. In AML, FTO is abnormally localized in the cytoplasm and exerts oncogenic effects by altering m^6^A demethylation [39]. ALKBH5, another m^6^A demethylase, is highly expressed in the testis, lungs, and germ cells, but weakly in cardiac and cerebral tissues [40, 41]. Unlike FTO, ALKBH5 has no activity toward m^6^A_m_ and can be induced under hypoxia conditions in tumors, promoting self-renewal of glioblastoma and breast CSCs [42].

### m^6^A readers

m^6^A readers are RNA-binding proteins that specifically recognize and bind to m^6^A-modified RNA molecules. The YTH domain-containing proteins, including YTH domain-containing 1–2 (YTHDC1-2) and YTH domain family 1–3 (YTHDF1–3), were the first identified m^6^A readers. YTHDC1 promotes RNA splicing and nuclear export, and YTHDC2 weakly binds m^6^A and promotes mRNA translation and degradation. The YTHDF proteins have three highly similar paralogues: YTHDF1 enhances mRNA translation, YTHDF2 promotes mRNA degradation, and YTHDF3 performs both functions [43]. Additionally, m^6^A can indirectly recruit RNA-binding proteins by remodeling RNA structure, a phenomenon known as the“m^6^A-switch” [15]. The insulin-like growth factor 2 mRNA binding protein (IGF2BP) family and heterogeneous nuclear ribonucleoprotein (HNRNP) family belong to this category. IGF2BP1-3 promote mRNA stability, HNRNPC/G facilitate RNA splicing, and HNRNPA2B1 promotes both RNA splicing and degradation [15].

## m^6^A and tumor neovascularization

### m^6^A and angiogenesis

Among m^6^A writers, METTL3 and METTL14 are primarily investigated for their roles in tumor angiogenesis (Table 1). Firstly, they facilitate the expression of angiogenic factors in various cancers. For example, METTL3 directly promotes the expression of hypoxia-inducible factor 1-alpha (HIF-1α), VEGFA and tyrosine kinase (TEK) in bladder cancer (BLCA) [44, 45]. Similarly, METTL14 induces the expression of basic leucine zipper ATF-like transcription factor 2 (BATF2), which indirectly upregulates VEGFA secretion and promotes angiogenesis in tongue squamous cell carcinoma (TSCC) [46]. Beyond VEGFA, METTL3 also regulates ECM components to modulate angiogenesis [47]. In HCC and prostate cancer, METTL3 stimulates angiogenesis by increasing the expression of MMP2 and MMP9 [48–50]. Moreover, in CRC, METTL3 enhances plasminogen activator, urokinase (PLAU) expression, which activates angiogenic factors stored in the ECM, thereby facilitating angiogenesis [51, 52]. Additionally, METTL3 regulates cell cycle-associated proteins. In gastric cancer (GC) and in head and neck squamous cell carcinoma (HNSCC), METTL3 upregulates centromere protein F (CENPF), ensuring an adequate blood supply for rapidly dividing tumor cells [53, 54]. METTL3 and METTL14 also affect metabolism and inflammation, indirectly regulating angiogenesis. In GC, METTL3 increases hepatoma-derived growth factor (HDGF) expression, promoting glycolysis, which subsequently contributes to angiogenesis and liver metastasis [55]. In RCC, METTL14 activates TNF receptor-associated factor 1 (TRAF1), and indirectly facilitates angiogenesis [56].

**Table 1:** Tumor neovascularization regulated by m^6^A methylation in different types of cancer.

Cancer type	m^6^A regulator	Target molecules	Effect on tumor neovascularization	Types of tumor neovascularization	Reference
Breast cancer	YTHDF3	EGFR and VEGFA	Positive	Angiogenesis	PMID: 33125861
BLCA	METTL3	VEGFA and TEK	Positive	Angiogenesis	PMID: 33681207
	METTL3	BIRC5	Positive	Angiogenesis	PMID: 35749893
	ALKBH5	lncBLACAT3	Negative	Angiogenesis	PMID: 37612524
CRC	METTL3	PLAU	Positive	Angiogenesis	PMID: 35567945
	METTL3	VEGFA and EphA2	Positive	VM	PMID: 35595748
	WTAP	VEGFA	Positive	Angiogenesis	PMID: 37428639
	ALKBH5	circ3823	Negative	Angiogenesis	PMID: 34172072
	YTHDF3	circ3823	Negative	Angiogenesis	PMID: 34172072
	IGF2BP2	Cyclin D1	Positive	Angiogenesis	PMID: 36230970
	IGF2BP3	Cyclin D1 and VEGF	Positive	Angiogenesis	PMID: 32993738
GC	METTL3	CENPF	Positive	Angiogenesis	PMID: 37256823
	METTL3	HDGF	Positive	Angiogenesis	PMID: 31582403
	METTL3	ADAMTS9	Positive	Angiogenesis	PMID: 35574388
	IGF2BP3	HIF-1α	Positive	Angiogenesis	PMID: 34621671
Glioma (including GBM)	METTL3	HOTAM1	Positive	VM	PMID: 36086906
	METTL3	BUD13	Positive	VM	PMID: 36463205
	METTL3	MMP2, CDH1, CDH2,FN1	Negative	VM	PMID: 35261810
HCC	METTL3	YAP1	Positive	VM	PMID: 32920668
	METTL3	FOXO3	Negative	Angiogenesis	PMID: 32368828
	YTHDF2	IL11 and SERPINE2	Negative	Angiogenesis	PMID: 31735169
HNSCC	METTL3	CDC25B	Positive	Angiogenesis	PMID: 35287752
ICC	FTO	TEAD2	Negative	Angiogenesis	PMID: 31143705
Lung cancer	METTL3	VEGFA	Positive	Angiogenesis	PMID: 37103476
	IGF2BP2	FLT4	Positive	Angiogenesis	PMID: 37353784
	IGF2BP2	TK1	Positive	Angiogenesis	PMID: 33758932
	YTHDC2	lncZNRD1-AS1	Negative	Angiogenesis	PMID: 36581942
MM	ALKBH5	SAV1	Positive	Angiogenesis	PMID: 35414790
Pancreatic cancer	METTL3	lncLIFR-AS1	Positive	Angiogenesis	PMID: 34658294
RCC	METTL14	TRAF1	Positive	Angiogenesis	PMID: 35538475
	FTO	VHL	Positive	Angiogenesis	PMID: 32817424
	YTHDF2	circPOLR2A	Negative	Angiogenesis	PMID: 35840930
TSCC	METTL14	BATF2	Positive	Angiogenesis	PMID: 35949109

*BLCA* bladder cancer, *CRC* colorectal cancer, *GBM* glioblastoma, *GC* gastric carcinoma, *HCC* hepatocellular carcinoma, *HNSCC* head and neck squamous cell carcinoma, *ICC* intrahepatic cholangiocarcinoma, *m*^*6*^*A* N6-methyladenosine, *MM* multiple myeloma, *RCC* renal cell carcinoma, *TSCC* tongue squamous cell carcinoma, *VM* vasculogenic mimicry.

The regulation of tumor angiogenesis by m^6^A erasers varies across different cancer types. In multiple myeloma (MM), ALKBH5 promotes angiogenesis by elevating salvador family WW domain-containing protein 1 (SAV1) expression and activating the Hippo pathway [57]. Conversely, in BLCA, ALKBH5 suppresses angiogenesis and hematogenous metastasis by inhibiting lncBLACAT3 expression, which inactivates the NF-κB pathway [58]. In RCC, FTO promotes angiogenesis by inhibiting von Hippel-Lindau tumor suppressor (VHL) expression, whereas in intrahepatic cholangiocarcinoma (ICC), FTO suppresses angiogenesis by inducing TEA domain transcription factor 2 (TEAD2) expression [59, 60]. These findings demonstrate that the effects of m^6^A erasers on angiogenesis are specific to the cancer type.

Correlations between tumor angiogenesis and m^6^A readers have been observed in both the IGF2BP and YTH domain-containing proteins, which exhibit opposing functions. The IGF2BP family exerts a pro-angiogenic effect by enhancing the stability of downstream genes. For instance, in lung cancer, IGF2BP2 increases the stability of fms-related tyrosine kinase 4 (*FLT4*; also known as VEGFR3) or thymidine kinase 1 (*TK1*) mRNA, leading to tumor angiogenesis and aggressiveness [61, 62]. Similarly, in CRC, IGF2BP2 and IGF2BP3 stabilize *cyclin D1* and *VEGF* mRNA, thereby promoting angiogenesis [63, 64]. In contrast, the YTH domain-containing proteins have been demonstrated to suppress tumor angiogenesis. In lung cancer, YTHDC2 enhances the translation efficiency of lncZNRD1-AS1, further suppressing angiogenesis and tumorigenesis through the miR-942/Tensin 1 axis [65]. Additionally, YTHDF2 inhibits angiogenesis in clear cell RCC (cRCC) and HCC by facilitating the degradation of target genes. In cRCC, YTHDF2 inhibits angiogenesis by increasing circPOLR2A degradation [66]. In HCC, it increases the degradation of IL11 and serpin family E member 2 (SERPINE2) and thus contributing to vascular normalization [67].

### m^6^A and vasculogenic mimicry

The well-studied m^6^A regulator METTL3, is associated with vasculogenic mimicry (VM) in CRC, glioma, and HCC [68–70]. In CRC, METTL3 promotes VM indirectly by targeting Eph receptor A2 (EphA2) and VEGFA, enhancing their stability through IGF2BP2 and IGF2BP3, respectively [69]. Elevated METTL3 levels in glioma contribute to VM through targeting HOXA transcript antisense RNA myeloid-specific 1 (HOTAIRM1) [70]. Similarly, in HCC, inhibition of METTL3 impairs VM-related tumor vasculature formation, indicating a positive correlation between m^6^A levels and VM [68]. These findings suggest that METTL3 regulates target genes at the translational level, ultimately inducing VM.

However, the effect of METTL3 on VM in glioblastomas (GBM) appears to be different. One study found that METTL3 enhances the stability of BUD13 homolog (BUD13), promoting the translation of cyclin-dependent kinase 12 (CDK12) and muscleblind-like splicing regulator 1 (MBNL1). This cascade results in the upregulation of MMP2 and laminin subunit gamma 2 (LAMC2), promoting VM [71]. Conversely, another study indicates that reduced METTL3 facilitates VM and is correlated with higher histopathological grade and lower overall survival [72]. The contradictory results may be attributed to differences in cell line selection and sample size. Further research is needed to clarify the specific role of m^6^A on VM in different tumors.

### m^6^A and stemness-associated factors

#### m^6^A and Oct4

Octamer-binding transcription factor 4 (*Oct4*), a member of the POU transcription factor family, is essential for stemness maintenance and differentiation of CSCs [73]. Recent studies have also linked it to angiogenesis [74]. Our previous research demonstrated that Oct4 regulates the differentiation of liver CSCs into tumor ECs [75]. YTHDF2 interacts with the 5′UTR of *Oct4* mRNA to increase its expression, thus maintaining stemness and promoting lung metastasis in HCC [76]. ALKBH5 is positively correlated with Oct4 in MM and non-small cell lung cancer. Suppression of ALKBH5 reduces Oct4 expression and inhibits CSC characteristics, suggesting that ALKBH5 may induce neovascularization through CSC-derived vasculogenesis manner in these tumors [57, 77].

#### m^6^A and Sox2

SRY-box transcription factor 2 (*Sox2*) is a transcription factor that is essential for the self-renewal and pluripotency of stem cells. It has been demonstrated that Sox2 is capable of promoting tumor neovascularization. In ESCC, Sox2 promotes angiogenesis by inducing suprabasin expression [78]. Furthermore, it contributes to VM in CRC [79]. On the other hand, tumor neovascularization increases Sox2 expression, which in turn helps to maintain the CSC phenotype. In skin tumors, CSCs are found in proximity to ECs and reside within a perivascular microenvironment [80]. Besides, in retinoblastoma, VEGF has been found to stimulate Sox2 expression and enhance tumor invasiveness [81]. These findings demonstrate a reciprocal relationship between Sox2 and tumor neovascularization, where both factors reinforce the aggressive behavior of the tumor.

Sox2 is a downstream target of METTL3, with IGF2BP2 recognizing methylated *Sox2* transcripts and preventing their degradation [82, 83]. METTL3 sustains Sox2 expression through an m^6^A-mediated mechanism, thereby maintaining stemness and metastasis in CRC [82]. In GBM, METTL3 binds to the 3′UTR of *Sox2* mRNA, thus maintaining stemness and radioresistance [83]. Additionally, ALKBH5 has been reported to facilitate Sox2 expression in lung cancer, MM and endometrial cancer [57, 84, 85]. For instance, in lung cancer, ALKBH5 counteracts YTHDF2-mediated degradation of Sox2 and thereby promoting tumor aggressiveness [84].

## m^6^A and tumor neovascularization-associated pathways

### m^6^A and VEGF

VEGF, an extensively studied angiogenic factor, is produced by various cell types. The VEGF family consists of six members, with VEGFA being the most critical for tumor neovascularization. VEGFR are categorized into three subtypes, with VEGFR1 and VEGFR2 being the most prevalent in vascular ECs. VEGFR1 is responsible for hematopoiesis, and VEGFR2 is involved in vasculogenesis and angiogenesis. VEGFR3, mainly expressed in lymphatic ECs, is associated with lymphangiogenesis [86]. When VEGF binds to VEGFR, it triggers TEK phosphorylation, activating intracellular signaling pathways that regulate the proliferation, migration, survival, and penetration of vascular ECs, ultimately leading to neovascularization [18]. Moreover, VEGF infulences tumor neovascularization thorugh various downstream signaling pathways, including PI3K/AKT, MAPK, PLC, and SRC [87].

In lung cancer, METTL3 binds to the A859 site within the internal ribosome entry site of VEGFA 5’UTR, recruiting YTHDC2/eIF4GI complex to promote VEGFA translation and increase its expression [88]. This promoting effect of METTL3 on VEGFA is also observed in CRC, pancreatic cancer and BLCA [45, 69, 89]. However, in sorafenib-resistant HCC, METTL3 exerts an opposite effect. Depletion of METTL3 increases the expression of VEGFA and other angiogenic factors [90]. This may result from the alterations in the recognition site of the m^6^A-modified target gene following drug resistance, which affecting their binding capacity. In RCC, FTO is mutually exclusive with VHL. Elevated FTO inhibits VHL expression and increases VEGFA secretion [59]. IGF2BP3 has been reported to promote angiogenesis by interacting with VEGFA in CRC and GC [63, 69, 91]. Notably, it is IGF2BP3, rather than other IGF2BP members, that specifically binds to VEGFA [69]. Further research is required to elucidate the selectivity of m^6^A readers.

### m^6^A and EGFR

Epidermal growth factor receptor (EGFR) is a membrane receptor on the surface of epidermal cells that belongs to the tyrosine kinase receptor family. Upon activation by EGF, it initiates tyrosine kinase activity and activates downstream signaling such as PI3K/AKT, MAPK, and JAK/STAT pathways, which regulate various biological processes [92].

In breast cancer with brain metastases, YTHDF3 enhances the translation of *EGFR* and *VEGFA* mRNA by binding to eukaryotic translation initiation factor 3 subunit A (eIF3a). Suppressing YTHDF3 reduces blood vessel density and impairs brain endothelial tube formation, thereby decreasing brain metastasis and prolonging survival [93]. Under hypoxic conditions, YTHDF2 is downregulated in HCC. However, when overexpressed, it binds to the 3’UTR of the *EGFR* mRNA and accelerates its degradation. Consequently, this process suppresses the MAPK/ERK pathway, thereby inhibiting cell proliferation and tumor growth [94].

### m^6^A and PI3K/AKT

The PI3K/AKT signaling pathway is crutial in cancer development and progression. PI3K consists of a regulatory subunit (p85) and a catalytic subunit (p110). When activated, PI3K converts PIP2 to PIP3, subsequently activating PDK1 and AKT, while PTEN can counteract these effects. Upon activation, AKT stimulates mTOR, which in turn phosphorylates downstream substrates and regulates diverse biological processes [95]. The PI3K/AKT pathway is widely involved in tumor neovascularization. For instance, the inactivation of the p110 subunit impedes functional vessel formation, thereby restraining tumor growth. Activated AKT in tumor ECs results in an increase in nitric oxide levels, forstering vascular permebility. Conversely, the deletion of PTEN delays pericyte maturation, resulting in defective vascular remodeling [96–98].

In CRC, METTL3 promotes VM by activating the PI3K/AKT and ERK1/2 pathways [69]. Similarly, in GC, METTL3 promotes tumor angiogenesis through the ADAM metallopeptidase with thrombospondin type 1 motif 9 (ADAMTS9)-mediated PI3K/AKT pathway [99]. In pancreatic cancer, METTL3 increases the stability of lncLIFR-AS1 and indirectly promotes VEGFA expression. This, in turn, activates the AKT/mTOR pathway and further promotes tumor progression [89]. Using a bioinformatics database, Chen et al. found that METTL3 also regulates the PI3K/AKT pathway in BLCA, silencing METTL3 exerts an inhibitory effect on angiogenesis [45]. Furthermore, METTL14 is upregulated in sunitinib-resistant RCC compared to sensitive ones. It increases *TRAF1* mRNA stability in an IGF2BP2-dependent manner, activating the AKT/mTOR/HIF-1α pathway and facilitating angiogenesis [56]. Similarly, in lung adenocarcinoma, IGF2BP2 upregulation in metastatic subpopulations is associated with a poor prognosis. It enhances *FLT4* mRNA stability and activates the PI3K/AKT pathway, thereby promoting angiogenesis [61].

### m^6^A and MAPK

MAPK, a serine-threonine protein kinase, regulates biological processes such as cell proliferation, differentiation, and migration. The MAPK family comprises ERK, p38, JNK, and BMK1, which represent four distinct MAPK pathways [100]. Among these, the Ras/Raf/MEK/ERK pathway has been extensively investigated and is closely linked to cancer [101].

Phosphatidylethanolamine binding protein 1 (PEBP1) inhibits the Raf/MEK/ERK pathway by binding to Raf and disrupting the Raf/MEK complex. In cRCC, YTHDF2 increases PEBP1 expression, leading to ERK pathway inactivation. Specifically, circPOLR2A facilitates the interaction between PEBP1 and ubiquitin protein ligase E3C (UBE3C), thereby promoting PEBP1 degradation. YTHDF2 negatively regulates circPOLR2A, resulting in ERK pathway inactivation and ultimately suppressing angiogenesis, metastasis, and tumor growth [66]. In GC, METTL3 upregulates CENPF expression through HNRNPA2B1. Elevated CENPF binds to focal adhesion kinase (FAK) and promotes its nuclear export, thereby activating the MAPK pathway and thus promoting angiogenesis and liver metastasis [53]. Similarly, METTL3, WTAP, and YTHDC1 are involved in MAPK pathway activation, enhancing tumor neovascularization in CRC [51, 69, 102].

### m^6^A and Hippo

The Hippo pathway, comprising FZD2, LATS1/2, SAV1, and YAP/TAZ, is essential for regulating CSC maintenance, angiogenesis, and drug resistance. FZD2 has been reported to induce VM and maintain stemness in HCC, while YAP is associated with angiogenesis and VM in various cancers [103, 104]. Recent studies indicate that the Hippo pathway is involved in m^6^A-mediated tumor neovascularization, especially in VM.

YAP exhibits widespread association with VM and can be mediated by m^6^A methylation. In HCC, METTL3 promotes *YAP* mRNA splicing and enhances its translation efficiency, thereby facilitating VM [68]. In pancreatic cancer, inhibition of YTHDF2 increases YAP expression and promotes EMT [105]. Using verteporfin, a YAP inhibitor, neovascularization is suppressed, as evidenced by reduced levels of angiopoietin-2 (Ang2), MMP2 and VE-cadherin [106]. Given that EMT serves as the mechanism underlying VM, it is speculated that YTHDF2 may inhibit VM in pancreatic cancer by suppressing the Hippo pathway. Similarly, in CRC, IGF2BP2 binds to *YAP* mRNA to promote its translation, and verteporfin reduces the number of cancer-associated fibroblasts, inhibiting angiogenesis and tumor progression [107–109]. Apart form YAP, SAV1 has been implicated in promoting stem cell phenotype and neovascularization in MM. Inhibition of ALKBH5 decreases SAV1 mRNA stability, suppresses the Hippo pathway and angiogenesis [57]. Considering the intimate connection among CSC, EMT and VM, it is worthwhile to investigate the role of m^6^A in regulating tumor neovascularization, especially in VM through the Hippo pathway.

### m^6^A and Wnt/β-catenin

The Wnt signaling pathway is important for regulating CSC maintenance and tumor metastasis, with three distinct pathways indentified: Wnt/β-catenin, Wnt/planar cell polarity, and Wnt/calcium. In the classical Wnt/β-catenin pathway, Wnt binds to the Frizzled receptor and activates the TCF/LEF transcription factor. Subsequently, TCF/LEF binds to β-catenin and promotes the transcription of various downstream genes [110].

**Fig. 2:**
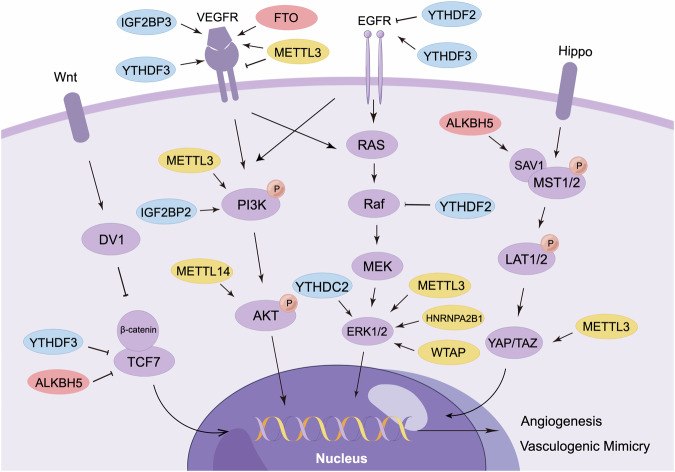
m^6^A regulators in various tumor neovascularization-associated signaling pathways. m^6^A “writers” (yellow circles), “erasers” (red circles), and “readers” (blue circles) selectively target specific signaling components, leading to the activation/inactivation of multiple intracellular signaling pathways (including Wnt/β-catenin, VEGFR, EGFR, Hippo, PI3K/AKT and MAPK pathways) associated with tumor neovascularization.

In CRC, upregulated circ3823 functions as a competing endogenous RNA, disrupting the suppressive effect of miR-30c-5p on TCF7, consequently activating the Wnt/β-catenin pathway. Suppression of YTHDF3 or ALKBH5 increases circ3823 expression, thus promoting angiogenesis, metastasis, and tumor growth [111] (Fig. 2).

## m^6^A in tumor vascular-immune crosstalk

Tumor angiogenesis creates an immunosuppressive microenvironment, aiding tumors in evading immune surveillance. Additionally, immunosuppressive cells can stimulate blood vessel formation, establishing abberrant communication between vascular and immune cells [112]. The synergistic effect of combining anti-angiogenic therapy with immunotherapy has demonstrated significant efficacy in treating various cancers, including RCC, HCC, GC, and endometrial cancer [113–116]. Recent studies highlight the role of m^6^A in regulating both tumor neovascularization and the immune microenvironment [117].

### m^6^A-mediated tumor vasculature effects on immune cells

**Fig. 3:**
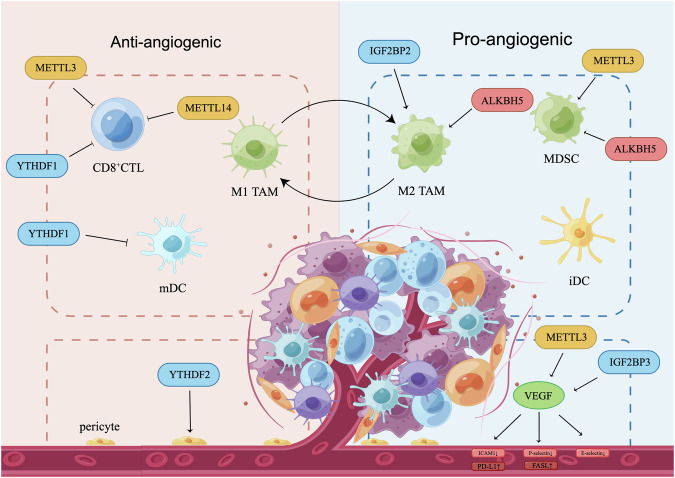
m^6^A regulators in vascular-immune crosstalk. Tumor neovascularization contributes to the establishment of an immunosuppressive tumor microenvironment, while immunosuppressive cells facilitate tumor angiogenesis through the secretion of pro-angiogenic factors. Immune effector cells like CD8 + CTL, M1 TAM, mDC contribute to the establishment of an anti-angiogenic TME, whereas immunosuppressive cells, such as M2-like TAM, MDSC, and iDC, promote angiogenesis. Meanwhile, VEGF reduces the expression of endothelial adhesion molecules (ICAM1, P-selectin, E-selectin) expression, inhibits DC maturation and CTL activation, increases the abundance of MDSC, consequently impedes the infiltration of immune cells. Furthermore, reduced pericyte coverage hinders blood vessel integrity and immune infiltration. m^6^A regulators play a role in modulating immune-vascular crosstalk by influencing various components such as immune cells, vascular-associated cells, angiogenic factors, and endothelial adhesion molecules. CTL cytotoxic T lymphocyte, FASL FAS ligand, iDC immature DC, M1 TAM M1-like tumor-associated macrophage, M2 TAM, M2-like tumor-associated macrophage, mDC mature DC, MDSC myeloid-derived suppressor cell, PD-L1 programmed cell death-1 ligand 1, ICAM1 intercellular adhesion molecule 1, VEGF vascular endothelial growth factor.

Immune cells extravasation into the TME requires adherence to ECs. However, ECs create an immune barrier by expressing programmed cell death-1 ligand 1 (PD-L1) and FAS ligand (FASL), as well as inhibiting adhesion factors such as intercellular adhesion molecule 1 (ICAM1), vascular cell adhesion molecule 1 (VCAM1), and P-selectin (Fig. 3) [118, 119]. Additionally, angiogenic factors like VEGF impede dendritic cells (DCs) maturation, impairing antigen presentation, suppressing tumor-specific cytotoxic T lymphocytes (CTLs) activation, or promoting the accumulation of immunosuppressive cells such as myeloid-derived suppressor cells (MDSCs) and regulatory T cells (Tregs) [120]. METTL3 and IGF2BP3 promote VEGF expression in various cancers, potentially contributing to the immunosuppressive TME [44, 63, 69, 88, 91].

Tumor blood vessels exhibit chaotic, leaky, and highly permeable characteristics, leading to increased interstitial fluid pressure that hampers immune cell infiltration. Inhomogeneous blood flow reduces perfusion and oxygenation, adversely impacting the delivery of anticancer drugs [121]. Pericytes are crucial for maintaining blood vessel structural integrity and have dual effects on tumor neovascularization. Generally, pericytes produce angiogenic factors that stimulate neovascularization [122, 123]. However, under certain conditions, increased pericyte coverage can normalize tumor vasculature [5]. In HCC, low levels of YTHDF2 due to hypoxia result in decreased pericyte coverage. Elevated YTHDF2 facilitates the degradation of IL11 and SERPINE2, reducing vascular density and permeability, thereby favorably impacting vascular normalization [67].

### m^6^A-mediated immune cell effects on tumor neovascularization

Immune cells regulate tumor angiogensis through the secretion of cytokines and chemokines, while m^6^A modification indirectly modulates this process by recruiting and activating immune cells. Tumor-associated macrophages (TAMs) exhibit distinct M1 or M2 phenotype, with M1-like TAMs promoting inflammation and inhibiting angiogenesis, and M2-like TAMs displaying an immunosuppressive phenotype that fosters angiogenesis [124]. ALKBH5 promotes M2-like TAMs recruitment in glioma [125]. In pancreatic cancer, lncPACERR promotes M2-like TAMs polarization via IGF2BP2, thereby driving tumor progression [126]. The impact of DCs on tumor angiogenesis depends on their maturation status. Mature DCs (mDCs) inhibit tumor angiogenesis, while immature DCs (iDCs) exhibit a pro-angiogenic effect [112, 120]. In GC, YTHDF1 deletion increases mDCs recruitment, implying a pro-angiogenic role of YTHDF1 [127]. In addition, other immunosuppressive cells such as MDSCs, Tregs, and Tie2-expressing macrophages also contribute to tumor angiogenesis [9, 112]. In CRC, METTL3 recruits MDSCs and promotes tumor growth [128]. Consistently, in cervical cancer, METTL3 positively correlates with CD33^+^ MDSCs and predicts unfavorable prognosis [129]. Conversely, in ICC, ALKBH5 decreases MDSC-like cell accumulation, enhances PD-L1 expression, thus facilitating immune infiltration [130].

Considering adaptive immunity, CD8^+^ CTLs release IFN-γ to normalize blood vessels, while helper T (Th) cells, including Th1, Th2, and Th17 subsets, actively participate in angiogenesis by releasing chemokines and activating M2-like TAMs [9, 112]. In CRC and melanoma, deletion of METTL3 and METTL14 increases CD8^+^ CTL levels and enhances response to anti-PD1 therapy [131]. Similarly, silencing of YTHDF1 in GC increases CD8^+^ CTL and mDCs proportions, thereby restoring sensitivity to immunotherapy (Table 2) [127].

**Table 2:** Fundamental functions of m^6^A regulators and their effect on tumor neovascularization.

Type	Regulator	Role	Regulation manner on tumor neovascularization	Reference
m^6^A writers	METTL3	Catalyze m^6^A modification	Promote angiogenesis in BLCA	PMID:35749893; PMID: 33681207
			Promote angiogenesis in CRC	PMID: 35567945
			Promote angiogenesis in GC	PMID:37256823; PMID: 31582403; PMID: 35574388
			Promote angiogenesis in HNSCC	PMID: 35287752
			Promote angiogenesis in lung cancer	PMID: 37103476
			Promote angiogenesis in pancreatic cancer	PMID: 34658294
			Inhibit angiogenesis in HCC	PMID: 32368828
			Promote VM in CRC	PMID: 35595748
			Promote VM in GBM	PMID: 36463205
			Promote VM in glioma	PMID: 36086906
			Promote VM in HCC	PMID: 32920668
			Inhibit VM in GBM	PMID: 35261810
			Recruit MDSCs in CRC	PMID: 35700773
			Recruit MDSCs in cervical cancer	PMID: 33059689
			Reduce CD8^+^CTL levels in CRC	PMID: 32964498
			Facilitate sorafenib resistance in HCC	PMID: 33222692; PMID: 32368828
			Facilitate apatinib resistance in HCC	PMID: 35503144
			Facilitate lenvatinib resistance in HCC	PMID: 36764493; PMID: 36898427; PMID: 36932115
	METTL14	Enhance the catalyze activity of METTL3	Promote angiogenesis in TSCC	PMID: 35949109
			Promote angiogenesis in RCC	PMID: 35538475
			Reduce CD8^+^CTL levels in CRC	PMID: 32964498
			Facilitate sunitinib resistance in RCC	PMID: 35538475
	WTAP	Help the localization of METTL3-METTL14 heterodimer	Promote angiogenesis in CRC	PMID: 37428639
			Facilitate lenvatinib resistance in HCC	PMID: 36932115
m^6^A erasers	FTO	Remove m^6^A modification	Promote angiogenesis in RCC	PMID: 32817424
			Inhibit angiogenesis in ICC	PMID: 31143705
	ALKBH5	Remove m^6^A modification	Promote angiogenesis in MM	PMID: 35414790
			Inhibit angiogenesis in CRC	PMID: 34172072
			Inhibit angiogenesis in BLCA	PMID: 37612524
			Recruit M2-like TAMs in glioma	PMID: 35444654
			Reduce MDSCs accumulation in ICC	PMID: 34301762
m^6^A readers	YTHDC1	Promote the splicing and nuclear export of RNA	Promote angiogenesis in CRC	PMID: 37428639
			facilitate sunitinib resistance in RCC	PMID: 35974388
	YTHDC2	Promote mRNA degradation and translation efficiency	Promote angiogenesis in lung cancer	PMID: 37103476; PMID: 36581942
	YTHDF1	Promote mRNA translation	Suppress mDCs recruitment and reduce CD8^+^CTLs proportion in GC	PMID: 35193930
	YTHDF2	Promote mRNA degradation	promote vascular normalization in HCC	PMID: 31735169
			inhibit angiogenesis in RCC	PMID: 35840930
			inhibit VM in RCC	PMID: 37037853
			Suppress pazopanib resistance in RCC	PMID: 37037853
	YTHDF3	Promote the translation and degradation of mRNA	Promote angiogenesis in breast cancer with brain metastases	PMID: 33125861
			Inhibit angiogenesis in CRC	PMID: 34172072
	IGF2BP2	stabilize mRNA and promote its translation	Promote angiogenesis in CRC	PMID: 36230970
			Promote angiogenesis in lung cancer	PMID: 37353784; PMID: 33758932
			Promote angiogenesis in RCC	PMID: 35538475
			Promote VM in CRC	PMID: 35595748
			Enhance M2-like TAMs polarization in pancreatic cancer	PMID: 35526050
			Facilitate sunitinib resistance in RCC	PMID: 35538475
	IGF2BP3	Stabilize mRNA and promote its translation	Promote angiogenesis in CRC	PMID: 32993738
			Promote angiogenesis in GC	PMID: 34621671
			Promote VM in CRC	PMID: 35595748
	HNRNPA2B1	Promote RNA splicing and degradation	Promote angiogenesis in GC	PMID: 37256823

*BLCA* bladder cancer, *CRC* colorectal cancer, *CTL* cytotoxic T lymphocyte, *GBM* glioblastoma, *GC* gastric carcinoma, *HCC* hepatocellular carcinoma, *HNSCC* head and neck squamous cell carcinoma, *ICC* intrahepatic cholangiocarcinoma, *m*^*6*^*A* N6-methyladenosine, *mDC* mature dendritic cell, *MDSC* myeloid-derived suppressor cell, MM multiple myeloma, *RCC* renal cell carcinoma, *TAM* tumor-associated macrophage, *TSCC* tongue squamous cell carcinoma, *VM* vasculogenic mimicry.

## m^6^A methylation in cancer therapy

### Clinical treatment prospects of new strategies

The advancement of targeted therapy strategies focused on m^6^A regulators is rapidly progressing, driven by their crucial regulatory functions and precise recognition abilities. Dysregulation of enzymes and binding proteins involved in RNA methylation has been linked to various human cancers, suggesting a promising novel approach for clinical intervention [34].

### m^6^A inhibitors

Recent studies indicate that inhibitors targeting RNA methylation regulatory factors have potential in cancer therapy. For instance, SAH and the broad-spectrum 2-OG oxygenase inhibitor IOX1 inhibit cancer development by targeting METTL3-METTL14 and ALKBH5, respectively [132]. Additionally, inhibitors like FB23-2, FG-2216/IOX3, Rhein, Entacapone, and meclofenamic acid inhibit FTO activity, preventing the self-renewal and tumorigenic properties of AML and GBM [132–134]. In mouse xenograft models, FTO inhibition resulted in reduced tumor growth and prolonged survival.

### Clinical trials and preclinical research

Ongoing clinical trials of m^6^A emphasize the potential of targeting m^6^A modifications in cancer treatment. STC-15, as an oral small molecule inhibitor targeting METTL3, is a first-in-class inhibitor of RNA modification. In November 2022, it advanced into Phase I clinical trials, representing the first RNA methyltransferase inhibitor to enter clinical development. STC-15 has demonstrated efficacy in suppressing AML or suppress tumor growth through anticancer immune responses, promising a novel avenue for cancer therapy [135].

Another promising candidate is STM2457, a highly selective METTL3 inhibitor with minimal effects on other methyltransferases, indicating its potential as a targeted cancer therapy. Preclinical research indicates that STM2457 specifically inhibits key stem cell populations in AML without significant toxicity to normal haematopoiesis, promoting cell differentiation, inducing apoptosis, and inhibiting tumor growth [136]. These findings support targeting m^6^A modification as a promising strategy for anticancer therapy.

### Potential of post-transcriptional modifications as biomarkers

Post-transcriptional modifications (PTMs) have been demonstrated to be associated with various human diseases. RNA modifications can be potential biomarkers for monitoring cancer progression through regulating mRNA stability, translation efficiency, and other RNA metabolic processes. Distinct modification patterns in different cancer types influence tumor cell behaviors such as proliferation, apoptosis, invasiveness, and metabolic activity, which makes them important indicators in cancer research. For instance, the m^5^C-based signature is an independent prognostic factor associated with immunotherapy efficacy and drug susceptibility in RCC [137]. The interaction network of m^6^A/m^5^C/m^1^A regulated genes is reported to assess the prognosis of HCC [138]. These imply the potential of RNA modification in clinical applications.

Clinically, detecting m^6^A often requires high-throughput sequencing (such as MeRIP-seq) and specific immunoprecipitation techniques [15]. These methods help clinicians analyze m^6^A levels in samples to assess tumor status and treatment response. As biomarkers, m^6^A modifications have the advantage of dynamically reflecting biological changes within tumor cells, allowing real-time monitoring of tumor progression and treatment response through non-invasive samples (such as blood) [139]. For instance, in gastrointestinal cancer, m^6^A levels were elevated compared to adjacent tissues and the serum of healthy individuals, and decreased post-surgery [140]. However, the dynamic and variable nature of m^6^A and other RNA modifications can complicate result interpretation. Moreover, the need for specialized techniques and equipment limits the widespread clinical application of m^6^A as a biomarker. Overall, research on RNA modifications as potential cancer biomarkers is still in its early stages, requiring further study to overcome current technical barriers and enhance their clinical accuracy and feasibility.

## Conclusions and perspectives

This review emphasizes the significant role of m^6^A modifications in tumor neovascularization. We aim to explore how m^6^A influences various modes of neovascularization and its interactions with multiple signaling pathways and components of the TME. By inhibiting key m^6^A writers or erasers, it may be possible to suppress pro-angiogenic factors, thereby reducing tumor vascularization and growth. This approach could potentially enhance the efficacy of existing treatments, such as VEGF inhibitors.

Although studies using m^6^A inhibitors to directly target tumor neovascularization are limited, growing evidence suggests that m^6^A is widely involved in anti-angiogenic resistance. Specifically, in HCC and RCC, both of which are characterized by high vascular density. Increased m^6^A levels contribute to resistance against sorafenib, apatinib, and lenvatinib in HCC. Under normoxic conditions, METTL3 increases resistance to sorafenib and lenvatinib through the Wnt/β-catenin pathway, while WTAP facilitates lenvatinib resistance under hypoxic conditions. Knockdown of METTL14 restores sensitivity to sorafenib by upregulating hepatocyte nuclear factor 3 gamma (HNF3γ). In RCC, downregulation of METTL14 enhances sensitivity to sunitinib by reducing TRAF1 expression. YTHDC1 increases sensitivity to sunitinib by inhibiting histone deacetylase 2 (HDAC2). Conversely, in pazopanib-resistant cRCC, YTHDF2 fails to recognize lncIGFL2AS1 for degradation, promoting drug resistance. These findings suggest targeting m^6^A might provide a novel approach to anti-angiogenic therapy. In conclusion, the regulatory role of m^6^A in tumor neovascularization is critical and warrants further investigation to develop potential treatment strategies. Additional research is needed to fully understand its clinical potential, especially its interactions with various pathways and immune cells. Despite the limited studies targeting tumor neovascularization with m^6^A inhibitors, growing evidence suggests that this approach may offer promising therapeutic potential.

## References

[1] Potente M, Gerhardt H, Carmeliet P. Basic and therapeutic aspects of angiogenesis. Cell. 2011;146:873–87. 21925313 10.1016/j.cell.2011.08.039

[2] Folkman J. Tumor angiogenesis: therapeutic implications. N Engl J Med. 1971;285:1182–6. 4938153 10.1056/NEJM197111182852108

[3] de Heer EC, Jalving M, Harris AL. HIFs, angiogenesis, and metabolism: elusive enemies in breast cancer. J Clin Investig. 2020;130:5074–87. 32870818 10.1172/JCI137552 PMC7524491

[4] Bergers G, Benjamin LE. Tumorigenesis and the angiogenic switch. Nat Rev Cancer. 2003;3:401–10. 12778130 10.1038/nrc1093

[5] Hanahan D, Weinberg RA. Hallmarks of cancer: the next generation. Cell. 2011;144:646–74. 21376230 10.1016/j.cell.2011.02.013

[6] Bergers G, Hanahan D. Modes of resistance to anti-angiogenic therapy. Nat Rev Cancer. 2008;8:592–603. 18650835 10.1038/nrc2442 PMC2874834

[7] Carmeliet P, Jain RK. Molecular mechanisms and clinical applications of angiogenesis. Nature. 2011;473:298–307. 21593862 10.1038/nature10144 PMC4049445

[8] Lugano R, Ramachandran M, Dimberg A. Tumor angiogenesis: causes, consequences, challenges and opportunities. Cell Mol Life Sci. 2020;77:1745–70. 31690961 10.1007/s00018-019-03351-7 PMC7190605

[9] De Palma M, Biziato D, Petrova TV. Microenvironmental regulation of tumour angiogenesis. Nat Rev Cancer. 2017;17:457–74. 28706266 10.1038/nrc.2017.51

[10] Hanahan D. Hallmarks of cancer: new dimensions. Cancer Discov. 2022;12:31–46. 35022204 10.1158/2159-8290.CD-21-1059

[11] Dawson MA, Kouzarides T. Cancer epigenetics: from mechanism to therapy. Cell. 2012;150:12–27. 22770212 10.1016/j.cell.2012.06.013

[12] An Y, Duan H. The role of m6A RNA methylation in cancer metabolism. Mol Cancer. 2022;21:14. 35022030 10.1186/s12943-022-01500-4 PMC8753874

[13] Song P, Tayier S, Cai Z, Jia G. RNA methylation in mammalian development and cancer. Cell Biol Toxicol. 2021;37:811–31. 34272618 10.1007/s10565-021-09627-8 PMC8599391

[14] Desrosiers R, Friderici K, Rottman F. Identification of methylated nucleosides in messenger RNA from Novikoff hepatoma cells. Proc Natl Acad Sci USA. 1974;71:3971–5. 4372599 10.1073/pnas.71.10.3971 PMC434308

[15] Zaccara S, Ries RJ, Jaffrey SR. Reading, writing and erasing mRNA methylation. Nat Rev Mol Cell Biol. 2019;20:608–24. 31520073 10.1038/s41580-019-0168-5

[16] Cao X, Geng Q, Fan D, Wang Q, Wang X, Zhang M, et al. m(6)A methylation: a process reshaping the tumour immune microenvironment and regulating immune evasion. Mol Cancer. 2023;22:42. 36859310 10.1186/s12943-022-01704-8 PMC9976403

[17] Uddin MB, Wang Z, Yang C. The m(6)A RNA methylation regulates oncogenic signaling pathways driving cell malignant transformation and carcinogenesis. Mol Cancer. 2021;20:61. 33814008 10.1186/s12943-021-01356-0 PMC8019509

[18] Pugh CW, Ratcliffe PJ. Regulation of angiogenesis by hypoxia: role of the HIF system. Nat Med. 2003;9:677–84. 12778166 10.1038/nm0603-677

[19] Maniotis AJ, Folberg R, Hess A, Seftor EA, Gardner LM, Pe’er J, et al. Vascular channel formation by human melanoma cells in vivo and in vitro: vasculogenic mimicry. Am J Pathol. 1999;155:739–52. 10487832 10.1016/S0002-9440(10)65173-5 PMC1866899

[20] Li X, Xue Y, Liu X, Zheng J, Shen S, Yang C, et al. ZRANB2/SNHG20/FOXK1 axis regulates vasculogenic mimicry formation in glioma. J Exp Clin Cancer Res. 2019;38:68. 30744670 10.1186/s13046-019-1073-7 PMC6371528

[21] Wang M, Zhao X, Zhu D, Liu T, Liang X, Liu F, et al. HIF-1alpha promoted vasculogenic mimicry formation in hepatocellular carcinoma through LOXL2 up-regulation in hypoxic tumor microenvironment. J Exp Clin Cancer Res. 2017;36:60. 28449718 10.1186/s13046-017-0533-1 PMC5408450

[22] Luo Y, Yang Z, Yu Y, Zhang P. HIF1alpha lactylation enhances KIAA1199 transcription to promote angiogenesis and vasculogenic mimicry in prostate cancer. Int J Biol Macromol. 2022;222:2225–43. 36209908 10.1016/j.ijbiomac.2022.10.014

[23] Yao XH, Ping YF, Bian XW. Contribution of cancer stem cells to tumor vasculogenic mimicry. Protein Cell. 2011;2:266–72. 21533771 10.1007/s13238-011-1041-2 PMC4875209

[24] Luo Q, Wang J, Zhao W, Peng Z, Liu X, Li B, et al. Vasculogenic mimicry in carcinogenesis and clinical applications. J Hematol Oncol. 2020;13:19. 32169087 10.1186/s13045-020-00858-6 PMC7071697

[25] Cai HP, Wang J, Xi SY, Ni XR, Chen YS, Yu YJ, et al. Tenascin-cmediated vasculogenic mimicry formation via regulation of MMP2/MMP9 in glioma. Cell Death Dis. 2019;10:879. 31754182 10.1038/s41419-019-2102-3 PMC6872754

[26] Liu J, Yue Y, Han D, Wang X, Fu Y, Zhang L, et al. A METTL3-METTL14 complex mediates mammalian nuclear RNA N6-adenosine methylation. Nat Chem Biol. 2014;10:93–5. 24316715 10.1038/nchembio.1432 PMC3911877

[27] Wang X, Feng J, Xue Y, Guan Z, Zhang D, Liu Z, et al. Structural basis of N(6)-adenosine methylation by the METTL3-METTL14 complex. Nature. 2016;534:575–8. 27281194 10.1038/nature18298

[28] Ping XL, Sun BF, Wang L, Xiao W, Yang X, Wang WJ, et al. Mammalian WTAP is a regulatory subunit of the RNA N6-methyladenosine methyltransferase. Cell Res. 2014;24:177–89. 24407421 10.1038/cr.2014.3 PMC3915904

[29] Patil DP, Chen CK, Pickering BF, Chow A, Jackson C, Guttman M, et al. m(6)A RNA methylation promotes XIST-mediated transcriptional repression. Nature. 2016;537:369–73. 27602518 10.1038/nature19342 PMC5509218

[30] Yue Y, Liu J, Cui X, Cao J, Luo G, Zhang Z, et al. VIRMA mediates preferential m(6)A mRNA methylation in 3’UTR and near stop codon and associates with alternative polyadenylation. Cell Discov. 2018;4:10. 29507755 10.1038/s41421-018-0019-0 PMC5826926

[31] Satterwhite ER, Mansfield KD. RNA methyltransferase METTL16: targets and function. Wiley Interdiscip Rev RNA. 2022;13:e1681. 34227247 10.1002/wrna.1681 PMC9286414

[32] Wang T, Kong S, Tao M, Ju S. The potential role of RNA N6-methyladenosine in cancer progression. Mol Cancer. 2020;19:88. 32398132 10.1186/s12943-020-01204-7 PMC7216508

[33] Lin H, Wang Y, Wang P, Long F, Wang T. Mutual regulation between N6-methyladenosine (m6A) modification and circular RNAs in cancer: impacts on therapeutic resistance. Mol Cancer. 2022;21:148. 35843942 10.1186/s12943-022-01620-x PMC9290271

[34] Chen XY, Zhang J, Zhu JS. The role of m(6)A RNA methylation in human cancer. Mol Cancer. 2019;18:103. 31142332 10.1186/s12943-019-1033-z PMC6540575

[35] Shi H, Wei J, He C. Where, when, and how: context-dependent functions of rna methylation writers, readers, and erasers. Mol Cell. 2019;74:640–50. 31100245 10.1016/j.molcel.2019.04.025 PMC6527355

[36] Deng X, Su R, Weng H, Huang H, Li Z, Chen J. RNA N(6)-methyladenosine modification in cancers: current status and perspectives. Cell Res. 2018;28:507–17. 29686311 10.1038/s41422-018-0034-6 PMC5951805

[37] Jia G, Fu Y, Zhao X, Dai Q, Zheng G, Yang Y, et al. N6-methyladenosine in nuclear RNA is a major substrate of the obesity-associated FTO. Nat Chem Biol. 2011;7:885–7. 22002720 10.1038/nchembio.687 PMC3218240

[38] Mauer J, Sindelar M, Despic V, Guez T, Hawley BR, Vasseur JJ, et al. FTO controls reversible m(6)Am RNA methylation during snRNA biogenesis. Nat Chem Biol. 2019;15:340–7. 30778204 10.1038/s41589-019-0231-8 PMC6984009

[39] Wei J, Liu F, Lu Z, Fei Q, Ai Y, He PC, et al. Differential m(6)A, m(6)A(m), and m(1)A demethylation mediated by FTO in the cell nucleus and cytoplasm. Mol Cell. 2018;71:973–85.e5. 30197295 10.1016/j.molcel.2018.08.011 PMC6151148

[40] Zheng G, Dahl JA, Niu Y, Fedorcsak P, Huang CM, Li CJ, et al. ALKBH5 is a mammalian RNA demethylase that impacts RNA metabolism and mouse fertility. Mol Cell. 2013;49:18–29. 23177736 10.1016/j.molcel.2012.10.015 PMC3646334

[41] Qu J, Yan H, Hou Y, Cao W, Liu Y, Zhang E, et al. RNA demethylase ALKBH5 in cancer: from mechanisms to therapeutic potential. J Hematol Oncol. 2022;15:8. 35063010 10.1186/s13045-022-01224-4 PMC8780705

[42] He L, Li H, Wu A, Peng Y, Shu G, Yin G. Functions of N6-methyladenosine and its role in cancer. Mol Cancer. 2019;18:176. 31801551 10.1186/s12943-019-1109-9 PMC6892141

[43] Jiang X, Liu B, Nie Z, Duan L, Xiong Q, Jin Z, et al. The role of m6A modification in the biological functions and diseases. Signal Transduct Target Ther. 2021;6:74. 33611339 10.1038/s41392-020-00450-x PMC7897327

[44] Liu H, Gu J, Huang Z, Han Z, Xin J, Yuan L, et al. Fine particulate matter induces METTL3-mediated m(6)A modification of BIRC5 mRNA in bladder cancer. J Hazard Mater. 2022;437:129310. 35749893 10.1016/j.jhazmat.2022.129310

[45] Wang G, Dai Y, Li K, Cheng M, Xiong G, Wang X, et al. Deficiency of Mettl3 in bladder cancer stem cells inhibits bladder cancer progression and angiogenesis. Front Cell Dev Biol. 2021;9:627706. 33681207 10.3389/fcell.2021.627706 PMC7930389

[46] Wen H, Tang J, Cui Y, Hou M, Zhou J. m6A modification-mediated BATF2 suppresses metastasis and angiogenesis of tongue squamous cell carcinoma through inhibiting VEGFA. Cell Cycle. 2023;22:100–16. 35949109 10.1080/15384101.2022.2109897 PMC9769451

[47] Sang QX. Complex role of matrix metalloproteinases in angiogenesis. Cell Res. 1998;8:171–7. 9791730 10.1038/cr.1998.17

[48] Liu J, Jiang K. METTL3-mediated maturation of miR-589-5p promotes the malignant development of liver cancer. J Cell Mol Med. 2022;26:2505–19. 35348293 10.1111/jcmm.16845 PMC9077310

[49] Chen YT, Xiang D, Zhao XY, Chu XY. Upregulation of lncRNA NIFK-AS1 in hepatocellular carcinoma by m(6)A methylation promotes disease progression and sorafenib resistance. Hum Cell. 2021;34:1800–11. 34374933 10.1007/s13577-021-00587-z

[50] Zheng Y, Qi F, Li L, Yu B, Cheng Y, Ge M, et al. LncNAP1L6 activates MMP pathway by stabilizing the m6A-modified NAP1L2 to promote malignant progression in prostate cancer. Cancer Gene Ther. 2023;30:209–18. 36195720 10.1038/s41417-022-00537-3 PMC9842505

[51] Yu T, Liu J, Wang Y, Chen W, Liu Z, Zhu L, et al. METTL3 promotes colorectal cancer metastasis by stabilizing PLAU mRNA in an m6A-dependent manner. Biochem Biophys Res Commun. 2022;614:9–16. 35567945 10.1016/j.bbrc.2022.04.141

[52] Smith HW, Marshall CJ. Regulation of cell signalling by uPAR. Nat Rev Mol Cell Biol. 2010;11:23–36. 20027185 10.1038/nrm2821

[53] Xu P, Yang J, Chen Z, Zhang X, Xia Y, Wang S, et al. N6-methyladenosine modification of CENPF mRNA facilitates gastric cancer metastasis via regulating FAK nuclear export. Cancer Commun. 2023;43:685–705. 10.1002/cac2.12443 PMC10259669 37256823

[54] Guo YQ, Wang Q, Wang JG, Gu YJ, Song PP, Wang SY, et al. METTL3 modulates m6A modification of CDC25B and promotes head and neck squamous cell carcinoma malignant progression. Exp Hematol Oncol. 2022;11:14. 35287752 10.1186/s40164-022-00256-3 PMC8919647

[55] Wang Q, Chen C, Ding Q, Zhao Y, Wang Z, Chen J, et al. METTL3-mediated m(6)A modification of HDGF mRNA promotes gastric cancer progression and has prognostic significance. Gut. 2020;69:1193–205. 31582403 10.1136/gutjnl-2019-319639

[56] Chen Y, Lu Z, Qi C, Yu C, Li Y, Huan W, et al. N(6)-methyladenosine-modified TRAF1 promotes sunitinib resistance by regulating apoptosis and angiogenesis in a METTL14-dependent manner in renal cell carcinoma. Mol Cancer. 2022;21:111. 35538475 10.1186/s12943-022-01549-1 PMC9087993

[57] Yu T, Yao L, Yin H, Teng Y, Hong M, Wu Q. ALKBH5 promotes multiple myeloma tumorigenicity through inducing m(6)A-demethylation of SAV1 mRNA and myeloma stem cell phenotype. Int J Biol Sci. 2022;18:2235–48. 35414790 10.7150/ijbs.64943 PMC8990482

[58] Xie J, Zhang H, Wang K, Ni J, Ma X, Khoury CJ, et al. M6A-mediated-upregulation of lncRNA BLACAT3 promotes bladder cancer angiogenesis and hematogenous metastasis through YBX3 nuclear shuttling and enhancing NCF2 transcription. Oncogene. 2023;42:2956–70. 37612524 10.1038/s41388-023-02814-3 PMC10541332

[59] Xiao Y, Thakkar KN, Zhao H, Broughton J, Li Y, Seoane JA, et al. The m(6)A RNA demethylase FTO is a HIF-independent synthetic lethal partner with the VHL tumor suppressor. Proc Natl Acad Sci USA. 2020;117:21441–9. 32817424 10.1073/pnas.2000516117 PMC7474618

[60] Rong ZX, Li Z, He JJ, Liu LY, Ren XX, Gao J, et al. Downregulation of fat mass and obesity associated (FTO) promotes the progression of intrahepatic cholangiocarcinoma. Front Oncol. 2019;9:369. 31143705 10.3389/fonc.2019.00369 PMC6521779

[61] Fang H, Sun Q, Zhou J, Zhang H, Song Q, Zhang H, et al. m(6)A methylation reader IGF2BP2 activates endothelial cells to promote angiogenesis and metastasis of lung adenocarcinoma. Mol Cancer. 2023;22:99. 37353784 10.1186/s12943-023-01791-1 PMC10288689

[62] Ma YS, Shi BW, Guo JH, Liu JB, Yang XL, Xin R, et al. microRNA-320b suppresses HNF4G and IGF2BP2 expression to inhibit angiogenesis and tumor growth of lung cancer. Carcinogenesis. 2021;42:762–71. 33758932 10.1093/carcin/bgab023

[63] Yang Z, Wang T, Wu D, Min Z, Tan J, Yu B. RNA N6-methyladenosine reader IGF2BP3 regulates cell cycle and angiogenesis in colon cancer. J Exp Clin Cancer Res. 2020;39:203. 32993738 10.1186/s13046-020-01714-8 PMC7523351

[64] Bian Y, Wang Y, Xu S, Gao Z, Li C, Fan Z, et al. m(6)A Modification of long non-coding RNA HNF1A-AS1 facilitates cell cycle progression in colorectal cancer via IGF2BP2-mediated CCND1 mRNA stabilization. Cells. 2022;11:3008. 36230970 10.3390/cells11193008 PMC9562639

[65] Wang J, Tan L, Yu X, Cao X, Jia B, Chen R, et al. lncRNA ZNRD1-AS1 promotes malignant lung cell proliferation, migration, and angiogenesis via the miR-942/TNS1 axis and is positively regulated by the m(6)A reader YTHDC2. Mol Cancer. 2022;21:229. 36581942 10.1186/s12943-022-01705-7 PMC9801573

[66] Xu Z, Chen S, Liu R, Chen H, Xu B, Xu W, et al. Circular RNA circPOLR2A promotes clear cell renal cell carcinoma progression by facilitating the UBE3C-induced ubiquitination of PEBP1 and, thereby, activating the ERK signaling pathway. Mol Cancer. 2022;21:146. 35840930 10.1186/s12943-022-01607-8 PMC9284792

[67] Hou J, Zhang H, Liu J, Zhao Z, Wang J, Lu Z, et al. YTHDF2 reduction fuels inflammation and vascular abnormalization in hepatocellular carcinoma. Mol Cancer. 2019;18:163. 31735169 10.1186/s12943-019-1082-3 PMC6859620

[68] Qiao K, Liu Y, Xu Z, Zhang H, Zhang H, Zhang C, et al. RNA m6A methylation promotes the formation of vasculogenic mimicry in hepatocellular carcinoma via Hippo pathway. Angiogenesis. 2021;24:83–96. 32920668 10.1007/s10456-020-09744-8

[69] Liu X, He H, Zhang F, Hu X, Bi F, Li K, et al. m6A methylated EphA2 and VEGFA through IGF2BP2/3 regulation promotes vasculogenic mimicry in colorectal cancer via PI3K/AKT and ERK1/2 signaling. Cell Death Dis. 2022;13:483. 35595748 10.1038/s41419-022-04950-2 PMC9122982

[70] Wu Z, Lin Y, Wei N. N6-methyladenosine-modified HOTAIRM1 promotes vasculogenic mimicry formation in glioma. Cancer Sci. 2022;114:129–41. 36086906 10.1111/cas.15578 PMC9807531

[71] Liu M, Ruan X, Liu X, Dong W, Wang D, Yang C, et al. The mechanism of BUD13 m6A methylation mediated MBNL1-phosphorylation by CDK12 regulating the vasculogenic mimicry in glioblastoma cells. Cell Death Dis. 2022;13:1017. 36463205 10.1038/s41419-022-05426-z PMC9719550

[72] Tao M, Li X, He L, Rong X, Wang H, Pan J, et al. Decreased RNA m(6)A methylation enhances the process of the epithelial mesenchymal transition and vasculogenic mimicry in glioblastoma. Am J Cancer Res. 2022;12:893–906. 35261810 PMC8899976

[73] Yang L, Shi P, Zhao G, Xu J, Peng W, Zhang J, et al. Targeting cancer stem cell pathways for cancer therapy. Signal Transduct Target Ther. 2020;5:8. 32296030 10.1038/s41392-020-0110-5 PMC7005297

[74] Hess DL, Kelly-Goss MR, Cherepanova OA, Nguyen AT, Baylis RA, Tkachenko S, et al. Perivascular cell-specific knockout of the stem cell pluripotency gene Oct4 inhibits angiogenesis. Nat Commun. 2019;10:967. 30814500 10.1038/s41467-019-08811-z PMC6393549

[75] Liu HL, Tang HT, Yang HL, Deng TT, Xu YP, Xu SQ, et al. Oct4 regulates the transition of cancer stem-like cells to tumor endothelial-like cells in human liver cancer. Front Cell Dev Biol. 2020;8:563316. 33102474 10.3389/fcell.2020.563316 PMC7554317

[76] Zhang C, Huang S, Zhuang H, Ruan S, Zhou Z, Huang K, et al. YTHDF2 promotes the liver cancer stem cell phenotype and cancer metastasis by regulating OCT4 expression via m6A RNA methylation. Oncogene. 2020;39:4507–18. 32366907 10.1038/s41388-020-1303-7

[77] Liu X, Wang Z, Yang Q, Hu X, Fu Q, Zhang X, et al. RNA demethylase ALKBH5 prevents lung cancer progression by regulating EMT and stemness via regulating p53. Front Oncol. 2022;12:858694. 35530319 10.3389/fonc.2022.858694 PMC9076132

[78] Takahashi K, Asano N, Imatani A, Kondo Y, Saito M, Takeuchi A, et al. Sox2 induces tumorigenesis and angiogenesis of early-stage esophageal squamous cell carcinoma through secretion of Suprabasin. Carcinogenesis. 2020;41:1543–52. 32055838 10.1093/carcin/bgaa014

[79] Chen J, Chen S, Zhuo L, Zhu Y, Zheng H. Regulation of cancer stem cell properties, angiogenesis, and vasculogenic mimicry by miR-450a-5p/SOX2 axis in colorectal cancer. Cell Death Dis. 2020;11:173. 32144236 10.1038/s41419-020-2361-z PMC7060320

[80] Beck B, Driessens G, Goossens S, Youssef KK, Kuchnio A, Caauwe A, et al. A vascular niche and a VEGF-Nrp1 loop regulate the initiation and stemness of skin tumours. Nature. 2011;478:399–403. 22012397 10.1038/nature10525

[81] Garcia JR, Gombos DS, Prospero CM, Ganapathy A, Penland RL, Chevez-Barrios P. Expression of angiogenic factors in invasive retinoblastoma tumors is associated with increase in tumor cells expressing stem cell marker Sox2. Arch Pathol Lab Med. 2015;139:1531–8. 26619025 10.5858/arpa.2014-0262-OA

[82] Li T, Hu PS, Zuo Z, Lin JF, Li X, Wu QN, et al. METTL3 facilitates tumor progression via an m(6)A-IGF2BP2-dependent mechanism in colorectal carcinoma. Mol Cancer. 2019;18:112. 31230592 10.1186/s12943-019-1038-7 PMC6589893

[83] Visvanathan A, Patil V, Arora A, Hegde AS, Arivazhagan A, Santosh V, et al. Essential role of METTL3-mediated m(6)A modification in glioma stem-like cells maintenance and radioresistance. Oncogene. 2018;37:522–33. 28991227 10.1038/onc.2017.351

[84] Zhang D, Ning J, Okon I, Zheng X, Satyanarayana G, Song P, et al. Suppression of m6A mRNA modification by DNA hypermethylated ALKBH5 aggravates the oncological behavior of KRAS mutation/LKB1 loss lung cancer. Cell Death Dis. 2021;12:518. 34016959 10.1038/s41419-021-03793-7 PMC8137886

[85] Chen G, Liu B, Yin S, Li S, Guo Y, Wang M, et al. Hypoxia induces an endometrial cancer stem-like cell phenotype via HIF-dependent demethylation of SOX2 mRNA. Oncogenesis. 2020;9:81. 32913192 10.1038/s41389-020-00265-z PMC7484801

[86] Apte RS, Chen DS, Ferrara N. VEGF in signaling and disease: beyond discovery and development. Cell. 2019;176:1248–64. 30849371 10.1016/j.cell.2019.01.021 PMC6410740

[87] Claesson-Welsh L, Welsh M. VEGFA and tumour angiogenesis. J Intern Med. 2013;273:114–27. 23216836 10.1111/joim.12019

[88] Zhang H, Zhou J, Li J, Wang Z, Chen Z, Lv Z, et al. N6-methyladenosine promotes translation of VEGFA to accelerate angiogenesis in lung cancer. Cancer Res. 2023;83:2208–25. 37103476 10.1158/0008-5472.CAN-22-2449

[89] Chen JQ, Tao YP, Hong YG, Li HF, Huang ZP, Xu XF, et al. M(6)A-mediated up-regulation of LncRNA LIFR-AS1 enhances the progression of pancreatic cancer via miRNA-150-5p/ VEGFA/Akt signaling. Cell Cycle. 2021;20:2507–18. 34658294 10.1080/15384101.2021.1991122 PMC8794522

[90] Lin Z, Niu Y, Wan A, Chen D, Liang H, Chen X, et al. RNA m(6) A methylation regulates sorafenib resistance in liver cancer through FOXO3-mediated autophagy. EMBO J. 2020;39:e103181. 32368828 10.15252/embj.2019103181 PMC7298296

[91] Jiang L, Li Y, He Y, Wei D, Yan L, Wen H. Knockdown of m6A reader IGF2BP3 inhibited hypoxia-induced cell migration and angiogenesis by regulating hypoxia inducible factor-1alpha in stomach cancer. Front Oncol. 2021;11:711207. 34621671 10.3389/fonc.2021.711207 PMC8490730

[92] Levantini E, Maroni G, Del Re M, Tenen DG. EGFR signaling pathway as therapeutic target in human cancers. Semin Cancer Biol. 2022;85:253–75. 35427766 10.1016/j.semcancer.2022.04.002

[93] Chang G, Shi L, Ye Y, Shi H, Zeng L, Tiwary S, et al. YTHDF3 induces the translation of m(6)A-enriched gene transcripts to promote breast cancer brain metastasis. Cancer Cell. 2020;38:857–71.e7. 33125861 10.1016/j.ccell.2020.10.004 PMC7738369

[94] Zhong L, Liao D, Zhang M, Zeng C, Li X, Zhang R, et al. YTHDF2 suppresses cell proliferation and growth via destabilizing the EGFR mRNA in hepatocellular carcinoma. Cancer Lett. 2019;442:252–61. 30423408 10.1016/j.canlet.2018.11.006

[95] Yang J, Nie J, Ma X, Wei Y, Peng Y, Wei X. Targeting PI3K in cancer: mechanisms and advances in clinical trials. Mol Cancer. 2019;18:26. 30782187 10.1186/s12943-019-0954-x PMC6379961

[96] Figueiredo AM, Villacampa P, Dieguez-Hurtado R, Jose Lozano J, Kobialka P, Cortazar AR, et al. Phosphoinositide 3-kinase-regulated pericyte maturation governs vascular remodeling. Circulation. 2020;142:688–704. 32466671 10.1161/CIRCULATIONAHA.119.042354

[97] Soler A, Serra H, Pearce W, Angulo A, Guillermet-Guibert J, Friedman LS, et al. Inhibition of the p110alpha isoform of PI 3-kinase stimulates nonfunctional tumor angiogenesis. J Exp Med. 2013;210:1937–45. 24043760 10.1084/jem.20121571 PMC3782054

[98] Karar J, Maity A. PI3K/AKT/mTOR pathway in angiogenesis. Front Mol Neurosci. 2011;4:51. 22144946 10.3389/fnmol.2011.00051 PMC3228996

[99] Wang N, Huo X, Zhang B, Chen X, Zhao S, Shi X, et al. METTL3-mediated ADAMTS9 suppression facilitates angiogenesis and carcinogenesis in gastric cancer. Front Oncol. 2022;12:861807. 35574388 10.3389/fonc.2022.861807 PMC9097454

[100] McCubrey JA, Lahair MM, Franklin RA. Reactive oxygen species-induced activation of the MAP kinase signaling pathways. Antioxid Redox Signal. 2006;8:1775–89. 16987031 10.1089/ars.2006.8.1775

[101] Ullah R, Yin Q, Snell AH, Wan L. RAF-MEK-ERK pathway in cancer evolution and treatment. Semin Cancer Biol. 2022;85:123–54. 33992782 10.1016/j.semcancer.2021.05.010

[102] Ye M, Chen J, Yu P, Hu C, Wang B, Bao J, et al. WTAP activates MAPK signaling through m6A methylation in VEGFA mRNA-mediated by YTHDC1 to promote colorectal cancer development. FASEB J. 2023;37:e23090. 37428639 10.1096/fj.202300344RRR

[103] Azad T, Ghahremani M, Yang X. The role of YAP and TAZ in angiogenesis and vascular mimicry. Cells. 2019;8:407. 31052445 10.3390/cells8050407 PMC6562567

[104] Ou H, Chen Z, Xiang L, Fang Y, Xu Y, Liu Q, et al. Frizzled 2-induced epithelial-mesenchymal transition correlates with vasculogenic mimicry, stemness, and Hippo signaling in hepatocellular carcinoma. Cancer Sci. 2019;110:1169–82. 30677195 10.1111/cas.13949 PMC6447835

[105] Chen J, Sun Y, Xu X, Wang D, He J, Zhou H, et al. YTH domain family 2 orchestrates epithelial-mesenchymal transition/proliferation dichotomy in pancreatic cancer cells. Cell Cycle. 2017;16:2259–71. 29135329 10.1080/15384101.2017.1380125 PMC5788481

[106] Wei H, Wang F, Wang Y, Li T, Xiu P, Zhong J, et al. Verteporfin suppresses cell survival, angiogenesis and vasculogenic mimicry of pancreatic ductal adenocarcinoma via disrupting the YAP-TEAD complex. Cancer Sci. 2017;108:478–87. 28002618 10.1111/cas.13138 PMC5378285

[107] Naktubtim C, Payuhakrit W, Uttarawichien T, Hassametto A, Suwannalert P. YAP, a novel target regulates F-actin rearrangement-associated CAFs transformation and promotes colorectal cancer cell progression. Biomed Pharmacother. 2022;155:113757. 36271545 10.1016/j.biopha.2022.113757

[108] Shen Y, Wang X, Liu Y, Singhal M, Gurkaslar C, Valls AF, et al. STAT3-YAP/TAZ signaling in endothelial cells promotes tumor angiogenesis. Sci Signal. 2021;14:eabj8393. 34874746 10.1126/scisignal.abj8393

[109] Cui J, Tian J, Wang W, He T, Li X, Gu C, et al. IGF2BP2 promotes the progression of colorectal cancer through a YAP-dependent mechanism. Cancer Sci. 2021;112:4087–99. 34309973 10.1111/cas.15083 PMC8486198

[110] Zhang Y, Wang X. Targeting the Wnt/beta-catenin signaling pathway in cancer. J Hematol Oncol. 2020;13:165. 33276800 10.1186/s13045-020-00990-3 PMC7716495

[111] Guo Y, Guo Y, Chen C, Fan D, Wu X, Zhao L, et al. Circ3823 contributes to growth, metastasis and angiogenesis of colorectal cancer: involvement of miR-30c-5p/TCF7 axis. Mol Cancer. 2021;20:93. 34172072 10.1186/s12943-021-01372-0 PMC8229759

[112] Lee WS, Yang H, Chon HJ, Kim C. Combination of anti-angiogenic therapy and immune checkpoint blockade normalizes vascular-immune crosstalk to potentiate cancer immunity. Exp Mol Med. 2020;52:1475–85. 32913278 10.1038/s12276-020-00500-y PMC8080646

[113] Rini BI, Plimack ER, Stus V, Gafanov R, Hawkins R, Nosov D, et al. Pembrolizumab plus Axitinib versus Sunitinib for Advanced Renal-Cell Carcinoma. N Engl J Med. 2019;380:1116–27. 30779529 10.1056/NEJMoa1816714

[114] Finn RS, Qin S, Ikeda M, Galle PR, Ducreux M, Kim TY, et al. Atezolizumab plus bevacizumab in unresectable hepatocellular carcinoma. N Engl J Med. 2020;382:1894–905. 32402160 10.1056/NEJMoa1915745

[115] Kawazoe A, Fukuoka S, Nakamura Y, Kuboki Y, Wakabayashi M, Nomura S, et al. Lenvatinib plus pembrolizumab in patients with advanced gastric cancer in the first-line or second-line setting (EPOC1706): an open-label, single-arm, phase 2 trial. Lancet Oncol. 2020;21:1057–65. 32589866 10.1016/S1470-2045(20)30271-0

[116] Makker V, Rasco D, Vogelzang NJ, Brose MS, Cohn AL, Mier J, et al. Lenvatinib plus pembrolizumab in patients with advanced endometrial cancer: an interim analysis of a multicentre, open-label, single-arm, phase 2 trial. Lancet Oncol. 2019;20:711–8. 30922731 10.1016/S1470-2045(19)30020-8 PMC11686814

[117] Li X, Ma S, Deng Y, Yi P, Yu J. Targeting the RNA m(6)A modification for cancer immunotherapy. Mol Cancer. 2022;21:76. 35296338 10.1186/s12943-022-01558-0 PMC8924732

[118] Huinen ZR, Huijbers EJM, van Beijnum JR, Nowak-Sliwinska P, Griffioen AW. Anti-angiogenic agents - overcoming tumour endothelial cell anergy and improving immunotherapy outcomes. Nat Rev Clin Oncol. 2021;18:527–40. 33833434 10.1038/s41571-021-00496-y

[119] Khan KA, Kerbel RS. Improving immunotherapy outcomes with anti-angiogenic treatments and vice versa. Nat Rev Clin Oncol. 2018;15:310–24. 29434333 10.1038/nrclinonc.2018.9

[120] Fukumura D, Kloepper J, Amoozgar Z, Duda DG, Jain RK. Enhancing cancer immunotherapy using antiangiogenics: opportunities and challenges. Nat Rev Clin Oncol. 2018;15:325–40. 29508855 10.1038/nrclinonc.2018.29 PMC5921900

[121] Viallard C, Larrivee B. Tumor angiogenesis and vascular normalization: alternative therapeutic targets. Angiogenesis. 2017;20:409–26. 28660302 10.1007/s10456-017-9562-9

[122] Cheng L, Huang Z, Zhou W, Wu Q, Donnola S, Liu JK, et al. Glioblastoma stem cells generate vascular pericytes to support vessel function and tumor growth. Cell. 2013;153:139–52. 23540695 10.1016/j.cell.2013.02.021 PMC3638263

[123] Zhou W, Chen C, Shi Y, Wu Q, Gimple RC, Fang X, et al. Targeting glioma stem cell-derived pericytes disrupts the blood-tumor barrier and improves chemotherapeutic efficacy. Cell Stem Cell. 2017;21:591–603.e4. 29100012 10.1016/j.stem.2017.10.002 PMC5687837

[124] Mantovani A, Sozzani S, Locati M, Allavena P, Sica A. Macrophage polarization: tumor-associated macrophages as a paradigm for polarized M2 mononuclear phagocytes. Trends Immunol. 2002;23:549–55. 12401408 10.1016/s1471-4906(02)02302-5

[125] Wei C, Wang B, Peng D, Zhang X, Li Z, Luo L, et al. Pan-cancer analysis shows That ALKBH5 is a potential prognostic and immunotherapeutic biomarker for multiple cancer types including gliomas. Front Immunol. 2022;13:849592. 35444654 10.3389/fimmu.2022.849592 PMC9013910

[126] Liu Y, Shi M, He X, Cao Y, Liu P, Li F, et al. LncRNA-PACERR induces pro-tumour macrophages via interacting with miR-671-3p and m6A-reader IGF2BP2 in pancreatic ductal adenocarcinoma. J Hematol Oncol. 2022;15:52. 35526050 10.1186/s13045-022-01272-w PMC9077921

[127] Bai X, Wong CC, Pan Y, Chen H, Liu W, Zhai J, et al. Loss of YTHDF1 in gastric tumors restores sensitivity to antitumor immunity by recruiting mature dendritic cells. J Immunother Cancer. 2022;10:e003663. 35193930 10.1136/jitc-2021-003663 PMC9066370

[128] Chen H, Pan Y, Zhou Q, Liang C, Wong CC, Zhou Y, et al. METTL3 inhibits antitumor immunity by targeting m(6)A-BHLHE41-CXCL1/CXCR2 axis to promote colorectal cancer. Gastroenterology. 2022;163:891–907. 35700773 10.1053/j.gastro.2022.06.024

[129] Ni HH, Zhang L, Huang H, Dai SQ, Li J. Connecting METTL3 and intratumoural CD33(+) MDSCs in predicting clinical outcome in cervical cancer. J Transl Med. 2020;18:393. 33059689 10.1186/s12967-020-02553-z PMC7565373

[130] Qiu X, Yang S, Wang S, Wu J, Zheng B, Wang K, et al. M(6)A demethylase ALKBH5 regulates PD-L1 expression and tumor immunoenvironment in intrahepatic cholangiocarcinoma. Cancer Res. 2021;81:4778–93. 34301762 10.1158/0008-5472.CAN-21-0468

[131] Wang L, Hui H, Agrawal K, Kang Y, Li N, Tang R, et al. m(6) A RNA methyltransferases METTL3/14 regulate immune responses to anti-PD-1 therapy. EMBO J. 2020;39:e104514. 32964498 10.15252/embj.2020104514 PMC7560214

[132] Geng Q, Cao X, Fan D, Wang Q, Wang X, Zhang M, et al. Potential medicinal value of N6-methyladenosine in autoimmune diseases and tumours. Br J Pharmacol. 2023. 10.1111/bph.16030 36624563

[133] Su R, Dong L, Li Y, Gao M, Han L, Wunderlich M, et al. Targeting FTO suppresses cancer stem cell maintenance and immune evasion. Cancer Cell. 2020;38:79–96.e11. 32531268 10.1016/j.ccell.2020.04.017 PMC7363590

[134] Xiao L, Li X, Mu Z, Zhou J, Zhou P, Xie C, et al. FTO inhibition enhances the antitumor effect of temozolomide by targeting MYC-miR-155/23a cluster-MXI1 feedback circuit in glioma. Cancer Res. 2020;80:3945–58. 32680921 10.1158/0008-5472.CAN-20-0132

[135] ST Ltd. Homepage. https://www.stormtherapeutics.com/clinical-trials/.

[136] Yankova E, Blackaby W, Albertella M, Rak J, De Braekeleer E, Tsagkogeorga G, et al. Small-molecule inhibition of METTL3 as a strategy against myeloid leukaemia. Nature. 2021;593:597–601. 33902106 10.1038/s41586-021-03536-w PMC7613134

[137] Zhang R, Gan W, Zong J, Hou Y, Zhou M, Yan Z, et al. Developing an m5C regulator-mediated RNA methylation modification signature to predict prognosis and immunotherapy efficacy in rectal cancer. Front Immunol. 2023;14:1054700. 36911744 10.3389/fimmu.2023.1054700 PMC9992543

[138] Li D, Li K, Zhang W, Yang KW, Mu DA, Jiang GJ, et al. The m6A/m5C/m1A regulated gene signature predicts the prognosis and correlates with the immune status of hepatocellular carcinoma. Front Immunol. 2022;13:918140. 35833147 10.3389/fimmu.2022.918140 PMC9272990

[139] Ge L, Zhang N, Chen Z, Song J, Wu Y, Li Z, et al. Level of N6-methyladenosine in peripheral blood RNA: a novel predictive biomarker for gastric cancer. Clin Chem. 2020;66:342–51. 32040577 10.1093/clinchem/hvz004

[140] Konno M, Koseki J, Asai A, Yamagata A, Shimamura T, Motooka D, et al. Distinct methylation levels of mature microRNAs in gastrointestinal cancers. Nat Commun. 2019;10:3888. 31467274 10.1038/s41467-019-11826-1 PMC6715669

